# Female Homicide in Italy in 2021: Different Criminological and Psychopathological Perspectives on the Phenomenon

**DOI:** 10.3390/ijerph19127537

**Published:** 2022-06-20

**Authors:** Claudio Terranova, Laura Marino, Francesco Pozzebon

**Affiliations:** Legal Medicine and Toxicology, Department of Cardiac, Thoracic, Vascular Sciences and Public Health, University of Padova, 35121 Padova, Italy; laura.marino.97@gmail.com (L.M.); francesco.pozzebon@studenti.unipd.it (F.P.)

**Keywords:** intentional female homicide, gender-based violence, elderly homicide, methods of homicide, victimology, homicide–suicide

## Abstract

The intentional homicide of female victims, which is most commonly perpetrated by intimate partners or family members, has been recognized in recent years as a matter of grave public concern that needs to be addressed from the cultural and judicial perspectives. To allow an in-depth criminological and psychopathological evaluation of female homicide in Italy in 2021 to be conducted, the authors performed a newspaper report analysis of the phenomenon. All female homicides that occurred in Italy in 2021 (*n* = 119) were included in the study. The analysis confirmed the low rate of female homicides in Italy when compared with other countries and also showed the phenomenon to be more complex than usually described. The highest rate of homicides was observed in elderly females when compared with other age groups, implying different criminological considerations and suggesting that gender-based violence may only explain some of the identified cases. The high incidence of suicide or attempted suicide among offenders, together with the high incidence of reported mental disorders in that population, suggests that a psychopathological perspective on the phenomenon of female homicide could help with the development and implementation of preventive strategies that focus on managing mental health at a territorial level and intervening in difficult domestic situations.

## 1. Introduction

In recent years, violence against women has been recognized as a matter of grave public concern that must be addressed from the cultural and judicial perspectives [1]. Many different forms of violence are of relevance in this regard, including sexual violence, sexual exploitation, maltreatment, stalking, attempted murder, and intentional homicide, which are all considered to potentially be indicative of gender-based violence [2]. Such actions are considered felonies in many countries, including Italy. The trends in terms of crimes against women can be measured using different approaches. For instance, national statistics systems may prove useful, although they can be influenced by the reporting and registration of the crimes, with the underestimation of the phenomenon being more likely in relation to certain crimes (e.g., sexual violence is typically underreported) [3]. 

Cases of intentional homicide, which is defined as an “unlawful death purposefully inflicted on a person by another person” [1], due to the seriousness of such offenses, are usually precisely reported. In 2017, a total of 87,000 women were killed worldwide, with 58% of them being killed by intimate partners or family members [4,5]. Moreover, in 2017, the crude global rates of female homicide, intimate-partner- or family-related homicide, and female intimate-partner homicide were reported to be 2.3, 1.3, and 0.8 per 100,000 of the female population, respectively. In the same year, the crude rate of female homicide in Europe (the region where the risk is known to be the lowest) was 0.7 per 100,000 of the female population [4]. The Italian crude rates for homicide in general, male homicide, and female homicide were 0.51, 0.63, and 0.39 per 100,000 of the population, respectively, with such values being quite low when compared with the rates of other Western countries [6]. 

Global, European, and Italian data all indicate that most female homicide victims are killed by intimate partners or family members [4,7]. Many possible explanations have been suggested for this finding, with most focusing on the psychopathological, sociological, and criminological perspectives on the phenomenon [8,9,10,11,12,13]. Gender inequality is considered the most important factor influencing gender-based violence; therefore, it is recognized as a significant aspect of intimate partner homicide. In addition, individual, cultural, and environmental factors (e.g., changes in the perpetrator’s behavior, the perpetrator’s perceived loss of control over the victim, the threat of divorce or separation, an abuser’s loss of employment, the presence of a child of the victim by a previous partner) may also modulate the tendency to engage in gender-based violence, meaning that such factors should be considered from a preventive perspective [3,14,15] when designing interventions at the cultural, social, and legislative levels [16,17]. 

Yet, while data at various levels suggest the prevalence of female homicide to be linked to gender-based violence, a more in-depth analysis of the phenomenon of female homicide could highlight integrative or alternative elements of the phenomenon, which may have important repercussions with regard to the preventive perspective. 

Data on female homicide in general and intimate-partner- or family-related homicide in particular are usually available within national statistics systems in different countries [4], which is why such data are often used when assessing the phenomenon. 

However, another possible approach when it comes to analyzing female homicide is the newspaper report analysis. This data source, which has been used in prior studies [18,19], offers the advantage of providing access to data not otherwise available, including those data deemed relevant from the psychopathological and criminological perspectives, which mostly concern the defendants. To mitigate the disadvantages associated with the characteristics of the data source (e.g., newspaper articles may be subject to biases and accuracies), a specific approach should be implemented when conducting the analysis. Then, this approach should allow a more in-depth assessment of the phenomenon of female homicide in relation to various aspects related to the victims and perpetrators of the crimes to be conducted. 

The aim of the present study was to perform a deeper criminological and psychopathological analysis of female homicide cases in Italy in 2021. The gathered data may prove useful in terms of correctly interpreting the phenomenon and suggesting appropriate cultural, judicial, and clinical interventions.

## 2. Materials and Methods

### 2.1. Data Collection

To identify female homicide cases, national newspapers published during the period from 1 January 2021 to 31 December 2021 were analyzed. The authors referred to female homicide cases and not to “feminicide”, as this term is used to define specifically the killing of a woman or a girl because of their gender [3]. The data source was chosen in order to collect data deemed relevant from the psychopathological and criminological perspectives and not otherwise available through the use of other sources, such as the national statistic system. The year 2021 was chosen as the most recent year for which data were available. Five newspapers with high circulations were analyzed: Corriere della Sera, Repubblica, La Stampa, Huffington Post Italian Edition, and Ansa. The following search terms were used (here translated from Italian into English) to search the newspapers for relevant articles: “female AND homicide”, “feminicide”, “homicide AND suicide”, “murdered AND arrested”, and “prosecution AND murderer”. Among the search terms, “feminicide” was also used because the press, at least in Italy, uses this word to describe a woman intentional homicide without any criminological assumption. 

### 2.2. Variables

The articles identified in the newspapers were read so as to collect data concerning the variables of relevance to both the victim and the perpetrator in each case, which are reported in Table 1 and Table 2, respectively. 

The variables of interest concerning the victim were as follows: personal data, circumstantial data, the relation between the victim and the murderer, and criminological data. The criminological data included the method of the homicide, the presence (or absence) of other victims, potential motives explaining the crime, and the presence (or absence) of victimological aspects regarding the deceased. The potential motives for the crimes were classified as follows: emotional motives (separation, divorce, conclusion of a relationship, jealousy, betrayal), money (property crime), drugs (history of drug dealing or use), a conflictual or chaotic family background (previous domestic violence), trivial reasons, or unknown motives. A victimological role was suggested by the presence of one or more of the following features: age (parties younger than 10 years of age or older than 65 years), a state of intoxication, the inability of the victim to defend herself due to weakness or a frail constitution, or the presence of a mental, neurological, or somatic disease.

In terms of the perpetrator or suspect, the following variables were of interest: personal data as well as criminological and psychopathological data. The criminological data included a generic criminal record, a previous history of violence toward the deceased female, and a history of crimes against the person. The psychopathological data included a diagnosis of a mental disorder or a substance use disorder as well as a history of attempted or completed suicide after the homicide. 

### 2.3. Consistency and Accuracy of the Data

To mitigate the disadvantages related to the use of newspapers as a data source (e.g., newspaper articles may be subject to biases and inaccuracies), the authors cross-checked the information presented in the articles for both consistency and accuracy. In particular, the data for this study (see variables in Table 1 and Table 2) were collected in parallel by L.M. and F.P., who reviewed newspapers independently of each other. An Excel spreadsheet was completed by the two authors. The third author (C.T.) reviewed the work for accuracy. 

To verify the collected information, the authors checked another source of data by accessing the website of the Italian Interior Minister [20] at the beginning of 2022. The Italian Interior Minister website provides the number of cases, the percentage of partner- or family-related homicide, and the cumulative description of the methods of homicide for 2021. Thus, this methodology allowed an analysis of all the cases of female homicide investigated by the Italian criminal courts in 2021 to be conducted.

### 2.4. Statistical Analysis

All the descriptive analyses in this study were performed using IBM SPSS Statistics 25.0 for Windows (Armonk, NY, USA) [21]. The analyses were performed separately for the victims and the perpetrators or suspects. The victims were subdivided into five groups according to their age. Direct standardization for the age groups (0–19 years, 20–34 years, 35–44 years, 45–64 years, and 65+ years) was performed in accordance with the European Standard Population 2013 data [22]. The descriptive statistics (means and proportions) were calculated, and the dichotomous variables were compared using the chi-squared test. A *p*-value of <0.05 was considered to be statistically significant. 

## 3. Results

A total of 119 female homicide cases were included in this study (mean age = 53.3 years; standard deviation (SD) = 21.4 years; median = 54 years). More specifically, there were 9 (7.5%) victims aged 0–19 years, 17 (14.2%) victims aged 20–34 years, 16 (13.4%) victims aged 35–44 years, 31 (26.0%) victims aged 45–64 years, and 46 (38.6%) victims aged 65 years or older. Of the 119 victims, 97 (81.5%) were Italian. 

**Figure 1:**
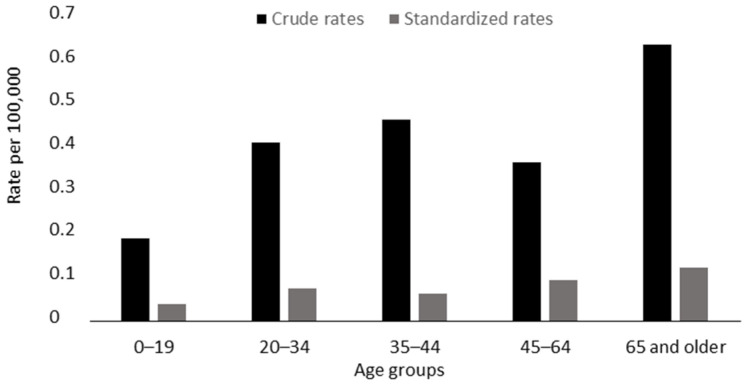
Crude and standardized homicide rates according to age groups.

The female homicide rate per 100,000 of the female Italian population and the homicide distribution in terms of the victims’ ages (after direct standardization) are reported in Table 3 and Figure 1, respectively. The victims’ marital status, parental status, education level, and employment status were not further considered due to the lack of relevant data. 

**Figure 2:**
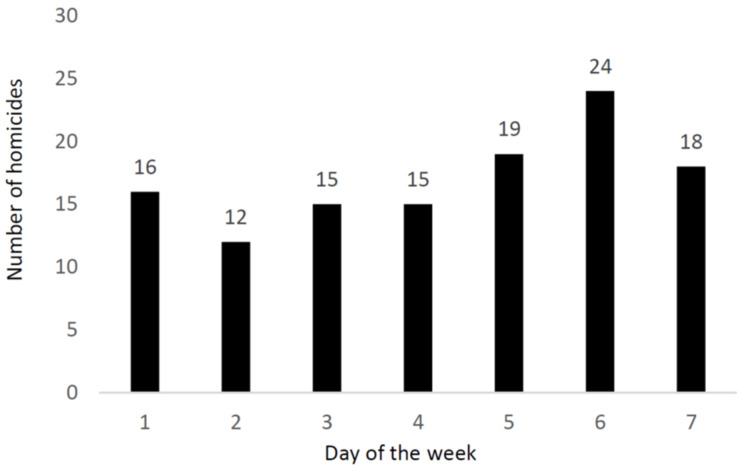
Number of homicides according to the day of the week. Note: 1 = Monday, 2 = Tuesday, 3 = Wednesday, 4 = Thursday, 5 = Friday, 6 = Saturday, 7 = Sunday.

**Figure 3:**
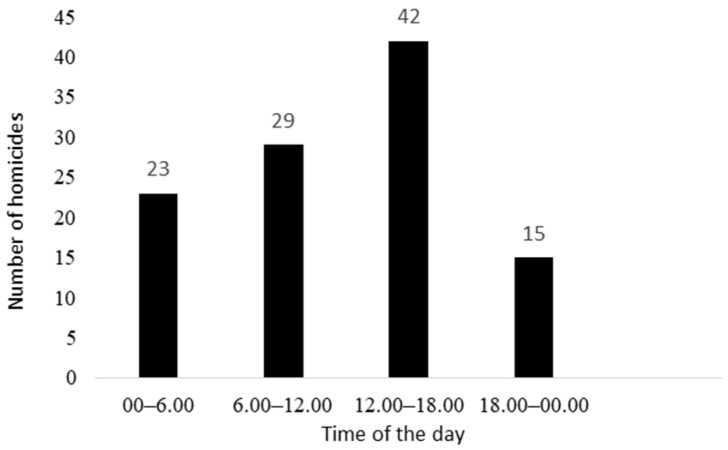
Number of homicides according to the time of the day.

The distributions of the homicides according to the day of the week and the time of the day are reported in Figure 2 and Figure 3, respectively. 

Some 61 (51.2%) of the homicides occurred on a Friday, Saturday, or Sunday. The majority of the homicides occurred in the victims’ home (87 out of 119 cases; 73.1%), followed by the street (11 cases; 9.2%), the victims’ workplace (5 cases; 4.2%), and other locations (16 cases; 13.4%). In 85 cases (71.4%), the weather was clear or partly cloudy, whereas in 34 cases (28.5%), the weather was rainy. 

In 75% of the cases (90 homicides), the homicide took place without any other parties being present. Intimate-partner- or family-related homicides of women and girls accounted for most of the sample (103 out of 119 cases; 86.5%). Moreover, 70 (58.8%) of the homicides were committed by a partner or ex-partner. Among the victims, 11 out of 119 were known to have a chronic neurologic disease. 

The most common methods of homicide were sharp-force injuries (52 cases; 43.6%), followed by firearm injuries (25 cases; 21%), asphyxia (17 cases; 14.2%), blunt-force injuries (14 cases; 11.7%), a combination of at least two methods (8 cases; 0.06), and falling from a height (1 case; 0.8%). Data in this regard were missing for two cases. In addition, in 17 cases (14.2%), there was at least one other victim. 

The most common method of homicide used by subjects aged 65 and over was firearm injuries (18 cases) followed by sharp-force injuries (12 case). The use of a firearm by a subject aged 65 and older was significantly more frequent than for the other age groups of perpetrators (18 out of 36 homicides committed by subjects 65 and over vs. 7 out of 80 homicides committed by perpetrators under the age of 65 years; *p* < 0.01). 

The alleged motives for the homicides were as follows: jealousy or betrayal in 14 cases (11.7%), separation or divorce in 39 cases (32.7%), “altruistic” reasons in 14 cases (11.7%), money in 19 cases (15.9%), psychopathology in 8 cases (6.7%), futile reasons in 7 cases (5.8%), unknown reasons in 20 cases (16.8%), and revenge in 6 cases (5.0%). Altruistic reasons were more common in murderers aged 65 and older than in the other age groups (10 homicides committed by subjects aged 65 and older vs. 4 homicides committed by perpetrators under the age of 65; *p* < 0.01). 

A total of 117 individual perpetrators were included in the analysis (mean age = 52.6 years; SD = 19.1 years; median = 50 years). Among the perpetrators, 109 were male (mean age = 53.4 years; SD = 19.0 years; median = 51.5 years) and 8 were female (mean age = 36.4 years; SD = 10.9 years; median = 40 years). In addition, one case involved three perpetrators, while another case involved two perpetrators.

The numbers of perpetrators with a generic criminal record, a history of violence toward the deceased female, a history of crimes against the person, a known psychiatric illness, a known substance use disorder, and a history of suicide or attempted suicide after the homicide are reported in Table 4. 

In total, 37 out of 117 (31.6%) individual perpetrators died by suicide after the homicide. In addition, 9.2% of homicides were followed by an attempted suicide. The perpetrators aged 65 years and over (36 individuals; 30.3%) died by suicide in 14 cases (38.8%), whereas the perpetrators under the age of 65 years (83 individuals; 69.7%) died by suicide in 23 cases (27.7%, *p* > 0.05). Notably, 15 out of 25 (60%) subjects that used a firearm to commit the crime died by suicide. This proportion was even higher in perpetrators aged 65 and over, using a firearm to commit the homicide, who attempted or died by suicide in 72% of cases. There were 46 victims aged 65 years or over (mean age = 75 years; SD = 7.1 years; median = 74) years, and they were killed by perpetrators with a mean age of 64.4 years (SD = 18.6 years; median = 72 years). The proportion of perpetrators aged 65 years and over was significant in the cases involving elderly female victims (26 vs. 46 elderly victims; 10 vs. 73 victims aged < 65; *p* < 0.001). In these cases, the homicide was followed by the completed suicide of the perpetrator in 14 (38.8%) instances, with no significant differences being found when compared with the cases involving other age groups. Attempted suicides (7 cases—19.4%) were more frequently described in the perpetrators aged 65 and over compared than in the other age groups (*p* = 0.01).

Among the 46 cases involving females aged 65 years or over, 42 (91.3%) involved the intimate-partner- or family-related killing of the victim. The offender in the cases involving victims aged 65 years and older was a partner or ex-partner in 25 out of 46 cases (54.3%) and a family member in 17 cases (36.4%). Only one of the cases involving this age group was considered to be a killing related to a crime. No kinship with the perpetrators or a relation with a crime was present in the last three cases. No statistically significant differences were observed in relation to the other age groups. In 11 cases (23.9%) involving a victim aged 65 years or older, the reason behind the homicide was deemed to be an “altruistic” motive. 

## 4. Discussion

The present study is innovative because it considers all female homicides that occurred in a Western country during 2021 by analyzing both the victim and the suspect from the criminological and psychopathological perspectives. 

The findings of the study confirmed that the female homicide rate in Italy in 2021 was low when compared with the rates in other European and non-European countries (e.g., rates in 2017 in Europe and in the Americas of 1.7 and 3.6, respectively) [4,23]. This low incidence is consistent with data concerning the rate of male homicide in Italy [3], which showed a progressive reduction starting from 1992 and due to the fall of Mafia-related homicides [3]. This form of homicide involved mostly males [3]. Furthermore, this result is not surprising due to the availability and use of firearms in Italy, which is lower than that observed in some countries (e.g., USA). Nevertheless, the reasons behind the low rate compared with other countries are certainly complex and would request a comparative study with a specific nation or group of countries, which is beyond the aim of the present study. The most worrying aspect of female homicide in Italy, as in other countries, concerns the lack of a downward trend equivalent to that observed with regard to male homicide. The fact that the murders most commonly occur in the context of a family or an intimate relationship is also a worrying aspect of female homicide [3].

The study findings also notably highlight that the highest female homicide rate (both crude and standardized) was found in relation to women aged 65 years and older. In fact, almost 40% of female homicide cases in Italy in 2021 involved elderly women, and these data are, in some ways, unexpected. Elderly homicide, independently of gender, differs from younger adult homicide [24,25] in terms of the characteristics of the offender and the possible motives [26]. Thus, the age of the victim should be carefully considered when interpreting the phenomenon of female homicide. Female homicide has traditionally been discussed and tackled in Italy by means of awareness campaigns and a regulatory approach focusing on a specific event, which usually takes place in the context of gender-based violence [27]. However, the interpretation of the phenomenon could differ for the elderly, who represent an important aspect of female homicide cases in Italy.

This study confirms that women in Italy are most commonly murdered by parties who are known to pose a danger and who are linked to their victim by an intimate relationship and/or family environment. Nonetheless, the phenomenon of female homicide is diversified in terms of its causes, as suggested by both the different ages of the victims and, more generally, the different characteristics of the victims. The victimological aspects of the murdered women could be related to the different dynamics of the event and the peculiar characteristics of the offender [24].

The killing of an elderly female by a partner or family member represented the prevalent situation in the 2021 Italian female homicide data. This result is not consistent with the data in the study by Kennedy et al. [28], who highlighted the presence of a stranger in almost a quarter of female homicide cases and noted crime to be a motive in the killing of approximately a third of elderly homicide cases. Our data may be explained by the low Italian homicide rate when compared with the rates of other crimes. The protective role of marriage described in other studies [29,30] is likewise not supported by the findings of this study, which is likely due to previously reported reasons (i.e., the low number of homicides when compared with other crimes).

Some of our cases may find as a possible explanation an “altruistic” motive even if most of the cases seem to be triggered by separation/divorce/betrayal. An “altruistic” homicide may be defined as a murder committed to relieve the real or imagined suffering of the victim. This is consistent with those cases involving elderly women in which the victim was affected by a chronic neurological disease. This finding is not unexpected, as a physical illness on the part of the victim, the perpetrator, or both is known to be associated with homicide–suicide cases in the elderly [31,32].

Among all the age groups in this study, the location of the homicide was predominantly the victim’s home, which is in line with the predominance of a close relationship between the victim and the perpetrator in the homicides. The fact that most of the homicides took place during the last three days of the week (Friday, Saturday, and Sunday) is consistent with the findings of previous studies [33,34].

Sharp-force injuries were found to be the most common methods of homicide in both this study and a prior study [35]. Such a finding is consistent with the results of previous studies conducted in countries with strict firearms legislation [36]. Unfortunately, the common use of sharp forces poses a difficulty when it comes to preventing such crimes, as the homicide is perpetrated using objects that are in common use. The data concerning the use of firearms, which was the second most common method of homicide in this study, are more interesting from the preventive perspective, as Italian legislation only allows a firearm to be used following the issuance of a license approved by a physician [37]. It is interesting to note that 72% of the perpetrators who committed homicide using a firearm were aged 65 years or older. The fact that 55% of these perpetrators died by suicide, while 72% attempted suicide or died by suicide, suggests the underestimation of the possible risk factors for violent behavior in the elderly with a license to possess a firearm.

Independently of the ages of the victim and the murderer, a suicide attempt or a completed suicide on the part of the perpetrator was described in a significant percentage of cases. These data are consistent with previous studies [38,39] that found a relation between interpersonal violence (IPV) and suicide, mostly in homicide–suicide cases. A history of IPV has also been found to be related to non-fatal suicidal ideation among the perpetrators of such crimes [40].

The data regarding the suicide of the perpetrator were confirmed in the cases involving elderly individuals in this study, which is consistent with the findings of prior studies specifically regarding this age group [41,42,43,44,45,46]. Yet, the hypothesis of correlation between IPV and suicide is not supported by our data in this specific group, suggesting the role of other risk factors. Indeed, untreated depression, another undiagnosed mental illness [47,48], social isolation, and a recent change in health status [41] could all be factors capable of triggering homicide–suicide. For these reasons, suicidal ideation should be investigated in those situations characterized at least by social isolation experienced by the elderly. Any type of violence, also dated, should be considered as a possible risk factor of mental distress [49,50].

Notably, elderly perpetrators attempted suicide more frequently than other age groups, and this result is, in some ways, unexpected [46,51]. Women and the young usually have more suicide attempts while suicide attempts among the elderly are usually characterized by lower impulsivity and higher lethal intent [46,51]; that is why suicide rates in males increase with age [48]. The data of this study could suggest that the elderly in our population were more vulnerable and affected by a mental distress than the other age groups.

A diagnosed mental disorder, including a substance use disorder (not counting attempted or completed suicide) was reported in 29.4% of the perpetrators (35 out of 119) in this study. These data should be more deeply analyzed, given that a causal relationship between psychiatric illness and criminality is difficult to assess. Certain psychiatric conditions are that not adequately treated [52] and/or substance use disorders [53] have previously been interpreted as possible factors leading to a higher risk of engaging in violent behavior than seen among the general population, although the causal relationship needs to be assessed in each case. The significant rate of mental disorders found in the present sample of perpetrators again suggests that the interpretation of the phenomenon of female homicide is complex due to the contribution of multiple factors related to the victim, the perpetrator, and their relationship.

### 4.1. Preventive Strategies

The fact that most of the homicides in this study involved females aged > 65 years confirms that the elderly represent an at-risk category when it comes to female homicide [54,55]. Thus, preventive strategies should focus on the risk factors present in this specific age group. Poor physical health, cognitive impairment, dependency on a relationship, and social isolation are all likely possible victimological risk factors. The implementation of social support and health assistance in relation to the elderly could provide an opportunity to intervene in a dangerous situation in some cases. It is possible that a valuable role could be played in this regard by general practitioners, who are usually in direct contact with elderly patients.

Mental illness on the part of the perpetrators was, in some way at least, involved in many of the female homicide cases in this study. The management of mental health at a territorial level could facilitate intervention in difficult situations via useful preventive actions. Suicidal ideation should be investigated in specific categories or social situations in which risk factors are present. Positive mental health, which manifests as high levels of subjective and psychological well-being [56], could help against suicide ideation/behavior, as suggested by some authors [57]. The elderly represents a specific group at risk, as showed by our study, particularly when a firearm has been used to commit the homicide. These data suggest the need to investigate the legal possession of a firearm in this age group particularly when signs of mental illness and/or a difficult familiar situation are present.

### 4.2. Strength and Limitations of the Study

The strengths of this study lie in the possibility of analyzing multiple variables related to both the perpetrator and the victim of the crime. These data are not available in other data sources in Italy. In addition, some of the variables were confirmed by a data source considered more reliable than newspaper reports, while most of the variables were unlikely to be misreported (e.g., the place of occurrence, date of the homicide, age of the victim, suicide or attempted suicide of the perpetrator).

The first limitation of the study concerns the utilized data source, which could include biases, as described above. The second limitation of the work is the inclusion of cases with a manner of death classified as homicide at the moment of data collection and not at the end of police investigation for all the cases. Even if it is difficult that an event classified as a homicide is then proved to be a suicide or an accidental death, this possibility may be not excluded. For the same reason, caution is requested when analyzing data referring to suicide reported by the press. This limitation should be considered when carrying out a comparative study with other countries where murders or suicides could be reported in a different way. Finally, the missing information not collected by the newspapers could have resulted in the underestimation of certain variables.

## 5. Conclusions

In conclusion, the findings of this study confirm the low incidence of female homicide in Italy when compared with other countries. Furthermore, the phenomenon is more complex on a criminological level than typically described. The highest rate of female homicides was observed among elderly women when compared with the other age groups. Importantly, elderly homicide is related to different criminological risk factors than homicide among other age groups.

The mental illness of the perpetrator is, in some way, related to female homicide, as suggested by the high rate of mental or substance use disorders as well as the high rates of attempted or completed suicides on the part of the perpetrators.

The criminological and psychopathological elements underlined in this study should be considered as the starting point for the development of preventive strategies. Prevention should focus not only on the awareness of gender-based violence as an aspect of homicide but also on risk factors involving the category of elderly people. The management of mental health at a territorial level could facilitate intervention in difficult situations via useful preventive actions.

## Figures and Tables

**Table 1:** Demographic variables concerning the victims.

VARIABLE	
PERSONAL DATA	
**Nationality**	Italian
	Not Italian
**Age**	
**Marital status**	Single
	Married
	Divorced
	Widow
**Education**	13 years–High School Degree
	Bachelor’s Degree
**Employment status**	Employed
	Freelance
	Unemployed
	Student
**CIRCUMSTANTIAL DATA**	
**Year, month, day of the week**	
**Time of occurrence**	0–6
	6–12
	12–18
	18–24
**Weather**	Rain–Fog–Snow
	Sunny–Clear Sky
**Place of occurrence**	Home
	Home of Third Party
	Public Place
	Street
	Workplace
	Other
**Presence of third party**	Yes
	No
**RELATION BETWEEN VICTIM AND MURDERER**	
**Relation**	Mother–Son
	Daughter–Parent
	Wife–Partner
**CRIMINOLOGICAL DATA**	
**Method**	Sharp-Force Injuries
	Firearms
	Asphyxia
	Blunt force
	Use of Fire
	More than one Method
	Falling from a Height
**Mass murder**	Yes
	No
**Potential motives explaining the crime**	

**Table 2:** Demographic variables concerning the murderers/suspects.

VARIABLE	
PERSONAL DATA	
**Nationality**	Italian
	Not Italian
**Age**	
**Marital status**	Single
	Married
	Divorced
	Widower/Widow
**Education**	13 years–High School Degree
	Bachelor’s Degree
**Employment status**	Employed
	Freelance
	Unemployed
	Student
**Criminological and psychopathological data**	
**Previous felonies**	
**Previous felonies against the victim**	
**Substance use disorder (SUD)**	
**Psychiatric disorder**	
**Substance use disorder**	
**Attempted suicide**	
**Death by suicide**	

**Table 3:** Homicide distribution in relation to the age of the victim (after direct standardization).

Age Group	Crude Rate	Homicide Rate (After Direct Standardization)
**0–19**	0.17	0.03
**20–34**	0.37	0.07
**35–44**	0.42	0.05
**45–64**	0.33	0.08
**65 and older**	0.58	0.11
**Total**	0.37	0.068

**Table 4:** Criminological and psychopathological variables concerning the murderers/suspects.

Variable	Author/Suspect N (%)
Total	122 (100%)
Generic criminal record	22 (18.0)
Precedents towards the victim	5 (4.0)
Crimes against the person	14 (11.4)
Psychiatric disorder	25 (20.4)
Substance use disorder (SUD)	13 (10.6)
Attempted suicide	11 (9.0)
Completed suicide	37 (30.3)

## Data Availability

The raw data required to reproduce the above findings cannot be shared at this time as the data also forms part of an ongoing study.

## References

[1] United Nations Office on Drugs and Crime (UNODC) (2014). Global Study on Homicide 2013.

[2] Alderton A., Henry N., Foster S., Badland H. (2020). Examining the relationship between urban liveability and gender-based violence: A systematic review. Health Place.

[3] Terranova C., Zen M. (2018). Women victims of intentional homicide in Italy: New insights comparing Italian trends to German and U.S. trends, 2008–2014. J. Forensic. Leg. Med..

[4] United Nations Office on Drugs and Crime (UNODC) Global Study on Homicide. Gender-Related Killing of Women and Girls, Vienna 2018.

[5] Smithey M., Thompson A. (2022). A Cross-National Examination of Global Gender Inequality and Femicide by Intimate Partners and Family Members. Violence Vict..

[6] Italian Interior Minister, 8 Marzo Donne Vittime di Violenza. https://www.interno.gov.it/it/stampa-e-comunicazione/dati-e-statistiche/omicidi-volontari-e-violenza-genere.

[7] McPhedran S., Eriksson L., Arnautovska U., Mazerolle P., Johnson H. (2022). Psychological Autopsy: A Method to Assist in the Identification of Risk and Protective Factors for Intimate Partner Femicide. Violence Against Women.

[8] Carabellese F., Tamma M., La Tegola D., Candelli C., Catanesi R. (2014). Women victims of violent partners: The Italian Situation amid culture and psychopathology. J. Forensic. Sci..

[9] Terranova C., Tucci M., Forza G., Barzon L., Palù G., Ferrara S.D. (2010). Alcohol dependence and glutamate decarboxylase gene polymorphisms in an Italian male population. Alcohol.

[10] Blanc A.K. (2001). The effect of power in sexual relationships on sexual and reproductive health: An examination of the evidence. Stud. Fam Plann..

[11] Jewkes R.K., Dunkle K., Nduna M., Shai N. (2010). Intimate partner violence, relationship power inequity, and incidence of HIV infections in young women in South Africa: A cohort study. Lancet.

[12] Santos-Hermoso J., González-Álvarez J.L., López-Ossorio J.J., García-Collantes Á., Alcázar-Córcoles M.Á. (2022). Psychopathic femicide: The influence of psychopathy on intimate partner homicide. J. Forensic. Sci..

[13] Carabellese F., Felthous A.R., Mandarelli G., Montalbò D., La Tegola D., Parmigiani G., Rossetto I., Franconi F., Ferretti F., Carabellese F. (2020). Women and Men who Committed Murder: Male/Female Psychopathic Homicides. J. Forensic. Sci..

[14] Adinkrah M. (2014). Intimate partner femicide-suicides in Ghana: Victims, offenders, and incident characteristics. Violence Against Women.

[15] Campbell J.C., Webster D., Koziol-McLain J., Block C., Campbell D., Curry M.A., Gary F., Glass N., McFarlane J., Sachs C. (2003). Risk factors for femicide in abusive relationships: Results rom a multi-site case control study. Am. J. Public Health.

[16] Mercy J.A. (2016). Global violence prevention: The time is now. Am. J. Prev. Med..

[17] Graham L.M., Macy R.J., Rizo C.F., Martin S.L. (2022). Explanatory Theories of Intimate Partner Homicide Perpetration: A Systematic Review. Trauma Violence Abuse.

[18] Lee K.A., Douglas E.M. (2021). An Exploratory Study of the Prosecution of Fatal Child Maltreatment: Criminal Charges Filed Against Presumed Perpetrators in the United States in 2017. Violence Vict..

[19] Saleh A.Z., Appelbaum P.S., Liu X., Scott Stroup T., Wall M. (2018). Deaths of people with mental illness during interactions with law enforcement. Int. J. Law Psychiatry.

[20] Italian Minister of the Interior. https://www.interno.gov.it/it/stampa-e-comunicazione/dati-e-statistiche/omicidi-volontari-e-violenza-genere.

[21] IBM (2017). IBM SPSS Statistics 25.0 for Windows.

[22] Eurostat, European Commission (2013). Revision of the European Standard Population—Report of Eurostat’s Task Force.

[23] United Nations Office on Drugs and Crime (UNODC) (2019). Global Study on Homicide 2019. UNODC: Vienna, Austria.

[24] Terranova C., Doro L., Zancaner S., Zampini T., Mazzarolo C., Bonvicini B., Viero A., Montisci M. (2020). Criminological and Medico-legal Aspects in Homicidal and Suicidal Sharp Force Fatalities. J. Forensic. Sci..

[25] United Nations Office on Drugs and Crime (UNODC) (2019). Homicide trends, patterns and criminal justice response. Global Study on Homicide.

[26] Swart L., Buthelezi S., Seedat M. (2019). The incidence and characteristics of homicides in elderly compared with non-elderly age groups in Johannesburg, South Africa. S. Afr. Med. J..

[27] Bellizzi S., Panu Napodano C.M., Lorettu L. (2022). Femicides in Europe: A tragedy beyond Italy. Int. J. Gynaecol. Obstet..

[28] Kennedy B., Bugeja L., Olivier J., Johnson M., Hua P., Koppel S., Ibrahim J.E. (2021). Epidemiology of Homicide in Community-Dwelling Older Adults: A Systematic Review and Meta-Analysis. Trauma Violence Abuse.

[29] Shackelford T.K. (2001). Cohabitation, marriage, and murder: Women killing by male romantic partners. Aggress. Behav..

[30] Shackelford T.K., Mouzos J. (2005). Partner killing by men in cohabiting and marital relationships: A comparative, cross-national analysis of data from Australia and the United States. J. Interpers. Violence.

[31] Sakuta T. (1995). A study of murder followed by suicide. Med. Law.

[32] Cohen D. (2000). Homicide-suicide in older persons. Psychiatr. Times.

[33] Baird A., While D., Flynn S., Ibrahim S., Kapur N., Appleby L., Shaw J. (2019). Do homicide rates increase during weekends andnational holidays?. J. Forens. Psychiatry Psychol..

[34] Nelson A.L., Bromley R.D.F., Thomas C.J. (2001). Idetifying micro-spatial and temporal patterns of violent crime and disorder in the British city centre. Appl. Geogr..

[35] Thomsen A.H., Hougen H.P., Villesen P., Brink O., Leth P.M. (2020). Sharp Force Homicide in Denmark 1992–2016. J. Forensic. Sci..

[36] Vassalini M., Verzeletti A., De Ferrari F. (2014). Sharp force injury fatalities: Aretrospective study (1982–2012) in Brescia (Italy). J. Forensic. Sci..

[37] Terranova C., Rocca G. (2016). Homicide committed by psychiatric patients: Psychiatrists’ liability in Italian law cases. Med. Sci. Law.

[38] Brown S., Seals J. (2019). Intimate partner problems and suicide: Are we missing the violence?. J. Inj. Violence Res..

[39] Kafka J.M., Moracco K.B.E., Taheri C., Young B.R., Graham L.M., Macy R.J., Proescholdbell S. (2022). Intimate partner violence victimization and perpetration as precursors to suicide. SSM Popul. Health.

[40] Wolford-Clevenger C., Brem M.J., Zapor H., Elmquist J., Stuart G.L. (2017). Prevalence, Severity, and Correlates of Suicidal Ideation Among Men and Women in Batterer Intervention Programs. Partn. Abuse.

[41] Bourget D., Gagné P., Whitehurst L. (2010). Domestic homicide and homicide-suicide: The older offender. J. Am. Acad. Psychiatry Law.

[42] Malphurs J.E., Cohen D. (2005). A statewide case-control study of spousal homicide-suicide in older persons. Am. J. Geriatr. Psychiatry.

[43] Cohen D., Llorente M., Eisdorfer C. (1998). Homicide-suicide in older persons. Am. J. Psychiatry.

[44] World Health Organization, Suicide. https://www.who.int/news-room/fact-sheets/detail/suicide.

[45] Mejías-Martín Y., Luna Del Castillo J.D., Rodríguez-Mejías C., Martí-García C., Valencia-Quintero J.P., García-Caro M.P. (2019). Factors Associated with Suicide Attempts and Suicides in the General Population of Andalusia (Spain). Int. J. Environ. Res. Public Health.

[46] Cano-Montalbán I., Quevedo-Blasco R. (2018). Sociodemographic Variables Most Associated with Suicidal Behaviour and Suicide Methods in Europe and America. A Systematic Review. Eur. J. Psychol. Appl. Leg Context.

[47] Nock M., Marzuk P., Jacobs D. (1999). Murder-suicide: Phenomenology and clinical implications. The Harvard Medical School Guide to Suicide Assessment and Intervention.

[48] Terranova C. (2022). Trends and Methods of Suicide in Italy, 1979 to 2016. SAGE Open.

[49] Sousa C.M.S., Mascarenhas M.D.M., Gomes K.R.O., Rodrigues M.T.P., Miranda C.E.S., Frota K.M.G. (2020). Suicidal ideation and associated factors among high school adolescents. Rev. Saude Publica.

[50] Gong M., Zhang S., Li W., Wang W., Wu R., Guo L., Lu C. (2020). Association between Childhood Maltreatment and Suicidal Ideation and Suicide Attempts among Chinese Adolescents: The Moderating Role of Depressive Symptoms. Int. J. Environ. Res. Public Health.

[51] Gramaglia C., Martelli M., Scotti L., Bestagini L., Gambaro E., Romero M., Zeppegno P. (2022). Attempted Suicide in the Older Adults: A Case Series from the Psychiatry Ward of the University Hospital Maggiore Della Carità, Novara, Italy. Front. Public Health.

[52] Bani-Fatemi A., Roy A., Dai N., Dada O., Adanty C., Kiruparajah L., Kolla N., Strauss J., Zai C., Graff A. (2020). Genome-wide association study of aggression and violence in schizophrenia. Neurosci. Lett..

[53] Arseneault L., Moffitt T., Caspi A., Taylor P.J., Silva P.A. (2000). Mental disorder and violence in a total birth cohort. Arch. Gen. Psychiatry.

[54] World Health Organization, Elder Abuse. https://www.who.int/news-room/fact-sheets/detail/elder-abuse.

[55] Terranova C., Bevilacqua G., Zen M., Montisci M. (2017). Crimes against the elderly in Italy, 2007-2014. J. Forensic. Leg Med..

[56] Keyes C.L. (2005). Mental illness and/or mental health? Investigating axioms of the complete state model of health. J. Consult. Clin. Psychol..

[57] Teismann T., Brailovskaia J., Margraf J. (2019). Positive mental health, positive affect and suicide ideation. Int. J. Clin. Health Psychol..

